# Spirituality and quality of life among Filipino women with breast cancer

**DOI:** 10.1017/S147895152510093X

**Published:** 2025-10-17

**Authors:** Gil Soriano, Alvin Hernandez

**Affiliations:** Department of Nursing, College of Allied Health, National University, Manila, Philippines

**Keywords:** breast cancer, quality of life, spirituality, Filipino women, meaning-in-life

## Abstract

**Objective:**

The study was conducted to determine the relationship between spirituality and the quality of life among women with breast cancer.

**Methods:**

This study utilized descriptive correlational research and a purposive sampling technique that involved women with breast cancer. Patients with breast cancer from particular breast cancer societies and organizations in Manila made up the sample. A total of 123 participants were included in the study. The Spiritual Index of Well-Being (SIWB) and the European Organization for Research and Treatment of Cancer Quality of Life Questionnaire were used to collect the needed data. Descriptive and inferential statistics were used to determine the relationship between spirituality and quality of life among women with breast cancer.

**Results:**

A high level of spirituality and quality of life were found among the participants. Overall, the mean score of the SIWB among the participants was 4.48 (±0.670), while the quality of life score was 62.6 (±10.9). A significant negative correlation was found between spirituality and quality of life (*r* = -0.127, *p* = 0.031), while significant positive correlations were noted between quality of life and self-efficacy (*r* = 0.683, *p* < 0.001) and life schemes or meaning in life (*r* = 0.704, *p* < 0.001).

**Significance of results:**

Although spirituality and quality of life had a negative correlation, the subscales of self-efficacy and life scheme had high positive correlations, indicating the complex dimensions of spirituality. In addition to providing coping strategies, spirituality offers patients the emotional, social, and existential support they need to deal with the unknowns of illness.

## Introduction

Cancer is one of the main causes of mortality worldwide. It is the leading cause of death among women in both high-income and middle-income countries. Among these, breast cancer is by far the most common cancer worldwide (Coccia 2013). Many Asian countries, including the Philippines, have the highest breast cancer mortality rate and the lowest mortality-to-incidence ratio. Filipino women face comparatively higher risks of developing breast cancer, with 1 out of 13 Filipino women expected to develop breast cancer in their lifetime, with an age-standardized rate of 47 per 100,000 women (Wu and Lee 2019). In the face of this life-threatening health condition, spirituality may be considered a valuable coping mechanism among patients (Ross et al. 2009).

Spirituality, according to Shaton et al. (2001), is the term used to describe human tendencies to explore life concepts with a yearning to grow oneself or connect with something greater than oneself. It is an inner power that enables one to see past self-interest and make sense of transcendental and experienced events. It is associated with positive health outcomes for women, from improved perception of health status (Musgrave and Allen 2002). Hence, spiritual needs are expectations and necessities for a person to find meaning, purpose, and value in their life (Büssing and Koenig 2010)

Reeves et al. (2011) stated that spirituality might be incorporated into patient care if desired. It is often linked with health care, spiritual or compassionate care, which involves serving the whole person – the physical, emotional, social, and spiritual. Such service is inherently a spiritual activity, forming the basis of meaning and purpose for many people (Puchalski 2001). This may help people engage in spiritual activities, leading to a higher quality of life (Cohen et al. 1995).

The WHO defines quality of life (QoL) as a person’s perspective on their life within the context of their culture and beliefs. It encompasses individual goals, hopes, and worries and is a subjective feeling of total life satisfaction based on perceived importance (Schalock 2004). Several aspects may impact this concept among patients with breast cancer, such as emotional distress. Most likely, after diagnosis, the concern of patients with cancer focuses on the treatability of the disease and the prognosis for their survival. However, once treatment begins, their worries might switch to the adverse effects of their treatment and how severe the procedure could be. Therefore, it is necessary to narrow the space between the patient’s hopes, aspirations, and actual events to improve the quality of life.

Recent studies highlight the importance of spirituality in shaping the health-related QoL among patients with cancer. Although results differ based on study design and the dimensions of spirituality assessed, a number of systematic reviews and meta-analyses confirm that spiritual or religiosity-informed interventions can enhance quality of life and lessen distress (Avcı and Çavuşoğlu 2025; Izgu et al. 2024; Nagy et al. 2024). Spirituality is a complex concept with multiple facets, and its constituent parts may have varying relationships with patient outcomes. While doctrinal or faith-based elements may have less pronounced or even context-dependent effects, meaning and peace, for instance, frequently exhibit considerable positive relationships with emotional and global QoL (Jafari et al., 2012; vos et al., 2015).

Spirituality is closely entwined with social and cultural systems in Southeast Asia, where patients frequently use communal behaviors, prayer, and family reliance as coping mechanisms for disease. Studies conducted in the Philippines specifically show how socioeconomic realities, including the cost of therapy, interact with spiritual and religious coping, which has a big impact on perceived QoL (Ahmadi et al. 2024; Santos et al. 2025).

In the Filipino cultural context, where spirituality and religiosity are profoundly ingrained in daily life and health practices, the results must also be interpreted. How patients view sickness and seek care is frequently influenced by their Catholic faith, prayer habits, and faith-based acceptance of suffering (Ahmadi et al. 2024; Doorenbos et al. 2011). Although social and emotional resources that improve resilience are provided by family-centeredness and the cultural value of bayanihan (mutual support), financial difficulties, which this study found to be the most bothersome symptom, intersect with spirituality in a variety of ways. For instance, a lack of socioeconomic means might exacerbate misery and lead to a conflict between practical challenges and spiritual acceptance, even while faith may provide existential meaning (Santos et al. 2025).

However, there is a dearth of literature exploring the spirituality and quality of life among patients living with breast cancer, as this is relatively new. The research was conducted to determine the relationship between spirituality and quality of life among Filipino women with breast cancer.

## Theoretical framework

A framework for understanding the complex relationship between spirituality and quality of life among women with breast cancer is provided by the Biopsychosocial-Spiritual Model. From a biological perspective, the model takes into account the physical effects that cancer and its therapies have on people (Engel 1980). Women’s interpretations and management of the physiological aspects of their sickness are influenced by their spirituality, which serves as a coping mechanism. In terms of psychology, spirituality is essential for fostering resilience and offering emotional support. Research has indicated that having a strong spiritual life is linked to improved mental health and a more optimistic outlook on life, even in the face of the difficulties presented by breast cancer (Lee et al., 2015). Furthermore, spiritual activities support existential well-being by assisting people in finding meaning and purpose despite receiving a diagnosis that drastically alters their lives (Vos et al. 2015)

Socially, the concept emphasizes the value of support systems and spiritual groups. Participation in these groups has been associated with increased social support, a sense of community, and decreased loneliness among breast cancer patients (Thuné-Boyle et al. 2006). A comprehensive understanding of the quality of life in the breast cancer setting necessitates addressing the spiritual dimension, as shown by the interconnectivity of these dimensions within the Biopsychosocial-Spiritual Model. A more thorough and patient-centered strategy that improves the general well-being of women with breast cancer requires the integration of spiritual care into healthcare interventions (Puchalski 2001).

The biological domain was indirectly represented in this study by the European Organization for Research and Treatment of Cancer Quality of Life Questionnaire (EORTC QLQ-C30), which examined the participants’ symptom load and treatment status. Using QoL functional measures, the psychological domain was recorded (e.g., emotional and cognitive functioning). The social domain was evaluated indirectly through items pertaining to role and social functioning and directly through demographic variables, including married and job status. The Spiritual Index of Well-Being (SIWB), which consists of two subscales, self-efficacy and life scheme, was the instrument used to specifically measure the spiritual domain. This study operationalized the framework into quantifiable components by assigning specific instruments to each dimension of Engel’s Biopsychosocial-Spiritual Model. This allowed for a more comprehensive analysis of the ways in which spirituality interacts with other factors that determine quality of life.

## Methodology

This study utilized a descriptive-correlational research as its main design to determine the quality of life and spirituality among women with breast cancer, thus, making it the most appropriate for this research. A purposive sampling technique was used to select the participants in the study. Patients with breast cancer from particular breast cancer societies and organizations in Manila made up the sample.

### Participants

Patients with breast cancer from particular breast cancer societies and organizations in Manila made up the sample. In order to be eligible to participate in the study, participants had to meet the following requirements: (1) be at least 45 years old; (2) have received a breast cancer diagnosis within the last five years; (3) be conscious, lucid, and oriented; (4) be willing to take part in the study; and (5) be actively receiving treatment for breast cancer, such as chemotherapy, surgery, or radiation therapy. Those who have completed chemotherapy and radiation therapy or are no longer receiving any treatment and who have experienced a breast cancer recurrence are excluded. According to Cohen (1988), a power of 0.95 required a sample size of 120; the alpha was set at 0.05, and the correlation medium effect size was set at 0.30. The total number of samples included in the study was 123.

### Instrumentation

The study utilized a three-part questionnaire which includes a demographic profile sheet, the SIWB scale and the EORTC QLQ.

#### Spiritual index of well-being (SIWB)

This tool was developed by Frey et al. (2005) and was intended to assess spiritual well-being and life’s meaning. It explained the patients’ views on spirituality and how it relates to their subjective well-being and health. Twelve items in all, divided into two domains, self-efficacy and life scheme, make up the instrument. Self-efficacy is assessed through six items (items 1, 2, 3, 4, 5, and 6) that identify the actions a person undertakes to overcome the challenges that could jeopardize their ability to operate and accomplish a goal. The life scheme, on the other hand, is assessed by six items (items 7, 8, 9, 10, 11, and 12), which evaluate a person’s feeling of coherence and the ability of a person to view a situation positively. The items in the instrument are rated using a 5-point Likert scale ranging from “strongly agree” (1 point), “agree” (2 points), “neither agree nor disagree” (3 points), disagree (4 points), and “strongly disagree (5 points). This was translated into Filipino by Soriano (2020). The Cronbach’s *α* coefficient for 12 items was 0.903, while the *α* coefficient for the self-efficacy scheme and life scheme was 0.864 and 0.889 (Soriano 2020).

#### EORTC quality of life questionnaire (QLQ) (Aaronson et al. 1993)

Based on the literature, the EORTC QLQ was the most widely used questionnaire for measuring QOL in individuals with breast cancer. It has a scale that includes symptoms like pain, fatigue, nausea and vomiting, dyspnea, sleep disturbance, constipation, diarrhea, appetite loss, and financial difficulties (13 items: nausea and vomiting, pain, dyspnea, sleep disturbance, appetite loss, constipation, diarrhea, and fatigue), as well as a functional scale that includes scores for social functioning, role functioning, physical functioning, cognitive functioning, and emotional functioning (15 items: strenuous activities, self-care, long/short walk, limitations at work, limitations in leisure, depression, worry, tension, and aggression). The global health/QOL scale uses modified 7-point linear analog scales for two of its items, but otherwise, all items are scored on a scale from 0 (not at all) to 4 (very lot). A high functional scale score indicates a high and healthy level of functioning, and a high global health status score indicates a high quality of life. On the other hand, a high symptom scale score indicates a high level of symptomatology or issues, which lowers QOL (Fayers et al. 2001).

### Data analysis

In order to analyze the data collected, IBM SPSS Statistics for Windows, Version 29.0 (IBM Corp., Armonk, NY, USA), frequency, percentage, weighted mean, standard deviation, and Pearson’s *r* correlation were used. Survey instruments with missing data were excluded in the data analysis.

### Ethical considerations

Before the study, the researchers obtained ethics clearance from the National University-Ethics Review Committee (NU-ERC) to ensure the study would conform to ethical standards with Protocol Number NU-ERC-2024-2T-09-IFRP. The participants were oriented regarding the study’s goal and objective, associated risks, and benefits of participation, and were encouraged to ask any questions regarding the study. Verbal and written consent were secured from the respondents who decided to participate.

## Results

Table 1 presents the demographic profile of the participants. The study samples included 123 women with a mean age of 51.4 years (±6.55), who were diagnosed in the past 2.50 years (±1.39), were predominantly Catholics, married, homemaker, completed high school, and were undergoing chemotherapy and radiation.

**Table 1.** Demographic Profile of the Participants (*n* = 123)

Characteristics	Frequency (*f*)	Percentage (%)	Mean (SD)
**Age (years)**			51.4 ± 6.55
**Year of diagnosis**			2.50 ± 1.39
**Religion**			
Catholic	100	81.3	
Non-Catholics	23	18.7	
**Civil status**			
Single	40	32.5	
Married	67	54.5	
Widowed	16	13	
**Employment**			
Employed	23	18.7	
Homemaker	57	46.34	
Retired	43	34.96	
**Educational attainment**			
Elementary	14	11.4	
High school	68	55.3	
College	41	33.3	
**Treatment (*n* = 151)^a^**			
Chemotherapy	39	25.83	
Chemotherapy and radiation	56	37.09	
Chemotherapy and surgery	46	30.46	
Chemotherapy, radiation and surgery	10	6.62	

a Participants could report multiple treatments; therefore, treatment categories are not mutually exclusive.

As illustrated in Table 2, the overall mean score of the SIWB among the participants was 4.48 (±0.670). The self-efficacy subscale, or the measure of an individual’s functional life self-efficacy, had a mean score of 4.50 (±0.702), while life scheme, or the one that assesses perception regarding making meaning in one’s life, had a mean score of 4.57 (±0.658).

**Table 2.** Spiritual Index of Well-Being and Self-transcendence among the participants

Variable	Mean	Standard Deviation
**Spiritual Index of Well-Being**	4.48	±0.670
Self-efficacy	4.5	±0.702
Life scheme	4.57	±0.658

Table 3 shows the mean score of all items in the quality of life among the participants. The mean quality of life score was 62.6 (±10.9). Of the five functional scales of the quality of life, the lowest score was cognitive functioning (31.4 ± 26.8), and the highest was physical functioning (73 ± 20.9). On the other hand, regarding the symptom scales, the most frequent complaint was financial difficulties, and the least was dyspnea.

**Table 3.** Mean score of all items in EORTC QLQ-C30 of the participants

Scales EORTC QLQ-C30	Mean	Standard Deviation
General health (QL)^a^	62.6	±10.9
**Functional scales**		
Physical functioning (PF)^a^	73	±20.9
Role functioning (RF)^a^	70.2	±24.3
Emotional functioning (EF)^a^	37.9	±25.5
Cognitive functioning (CF)^a^	31.4	±26.8
Social functioning (SF)^a^	33.5	±25.4
**Symptoms scales and items**		
Fatigue (FA)^b^	34.8	±21.5
Nausea and vomiting (NV)^b^	27.4	±23
Pain (PA)^b^	37.1	±24.9
Dyspnea (DY)^b^	24.4	±26
Insomnia (SL)^b^	32.2	±28
Appetite loss (AP)^b^	33.3	±25.6
Constipation (CO)^b^	31.7	±28.9
Diarrhea (DI)^b^	26.3	±29
Financial difficulties (FI)^b^	49.1	±27.8

EORTC QLQ = European Organization for Research and Treatment of Cancer Quality of Life Questionnaire.

a The higher the score on functional scales, the better the functionality.

b The higher the score on symptom scales, the greater the symptomatology.

Table 4 presents the relationship between spirituality and the composite scores of the quality of life index among the participants. Based on the results, a significant negative correlation exists between spirituality and quality of life (*r* coefficient of −0.127; *p*-value of 0.031). Further, significant positive correlations were also noted between quality of life and self-efficacy life (*r* = 0.683; *p* = <0.001) as well as life scheme (*r* = 704; *p* = < 0.001).

**Table 4.** Relationship between spiritual index of well-being and EORTC QLQ-C30

Variable	*r*	*p*-value
**Spiritual Index of Well-Being**	−0.127	0.031*
Self-efficacy	0.683	<0.001*
Life scheme	0.704	<0.001*

EORTC QLQ = European Organization for Research and Treatment of Cancer Quality of Life Questionnaire.

* *p*-value is significant at 0.05 level.

## Discussion

The results of this study emphasize the high levels of spiritual well-being among the participants, which is consistent with existing literature, which shows that spirituality is an important coping strategy for those dealing with life-threatening illnesses. By giving people a feeling of purpose and resilience, spirituality enables people to cope with fear, loss of control, and mortality (Koenig 2012; Puchalski 2001). Spirituality is an important aspect of how patients with breast cancer perceive their experiences and manage their emotional and physical difficulties (Ramey 2005). Additionally, it is linked to a more positive view of life, which is especially pertinent when it comes to patients with breast cancer, who frequently struggle with existential questions and the purpose of their suffering (Vos et al., 2017).

In terms of quality of life, the participants reported a high quality of life with a mean score of 62.6. The result was supported by the findings of Brandão et al. (2021), wherein Brazilian women with breast cancer reported a QoL score of 88%, and by Al-Natour et al. (2017) among Jordanian women. However, this was contradicted by the study of Jafari et al. (2012), who reported a mean score of 28.14. The difference in the results may be attributed to cultural differences among these countries. The high score among Filipino women may be due to the culture of strong family and social support. Strong familial ties and social support networks based on cultural customs like bayanihan (community unity) may help to explain the comparatively high QoL ratings reported by Filipino women. This assertion is supported by Doorenbos et al. (2011) who noted that family-centered caregiving influenced end-of-life coping in cancer patients, while de Guzman et al. (2009) discovered that Filipino elders derived resilience from family interdependence. According to more recent research, the resilience of Filipino patients with cancer is increased by their religiosity, group support, and meaning-making activities (Ahmadi et al. 2024). When someone in the family is sick, family members are expected to help (Luckman 2000). In turn, they develop a sense of obligation to care for one another (Nishimoto and Foley 2001). According to these findings, spirituality is not just an individual phenomenon in the Philippines but rather a family and community-based one, which may lessen the psychological effects of disease even in the face of material or physical hardship.

Pain, exhaustion, and appetite loss were among the most reported symptoms, while cognitive functioning was the most common in functional domains. According to Lee et al. (2015), these symptoms significantly affect patients’ quality of life and are frequently linked to cancer therapy and disease progression. This is in line with Hsu et al. (2017), who emphasized that the decreased quality of life of patients with breast cancer is largely caused by physical discomfort and adverse treatment effects. However, it can be noted that financial difficulties had the highest score in terms of symptom scales among the participants, outweighing physical complaints like exhaustion and pain. This result highlights the fact that the financial impact of continued treatment for breast cancer in this population goes beyond physical suffering. This means that the burden of disease lies not on the physical symptoms of breast cancer but on the expenses incurred while seeking treatment. This is consistent with research showing “financial toxicity” in cancer care (Zafar and Abernethy 2013) and is a frequently disregarded factor in determining quality of life for cancer survivors (Ahmadi et al. 2024). According to Fernandez and Ting (2023), financial struggles in the Philippines, where treatment costs can range from PHP 120,000 ($2,000 USD) to over PHP 1 million ($17,000 USD), might worsen psychological stress and have an impact on coping mechanisms, such as turning to spirituality. Therefore, integrating financial support networks with spiritual and psychosocial care may be essential to enhancing overall quality of life.

Self-efficacy and life scheme, which are the two main dimensions of spirituality, showed a positive correlation with QoL, indicating that these elements of spirituality may have a direct impact on well-being. According to Frey et al. (2005), self-efficacy is a person’s confidence in their ability to manage life’s obstacles. A higher level of self-efficacy can help cancer patients cope better and feel less burdened by their symptoms (Sawatzky et al. 2018). On the other hand, the life scheme dimension may help patients understand their events in a way that supports existential well-being (Frey et al. 2005).

An important finding in the study reveals a significant negative correlation between spirituality and quality of life. Although this finding deviates from a large portion of the literature, there are a number of possible explanations. First, it appears that spirituality does not impair QoL; rather, it may increase it as a coping strategy for individuals with poorer QoL (Frankl 1985; Jetan et al. 2023). Second, in societies where spiritual views of suffering are common, spirituality and discomfort may coexist, reflecting a search for purpose in hardship rather than the relief of pain (Ahmadi et al. 2024; Doorenbos et al. 2011).

When someone is faced with a life-threatening disease, their feeling of control and normalcy might be upset, which can lead to a desire for purpose. According to Frankl (1985), spirituality provides a framework for understanding pain, and individuals frequently find solace in the idea that their experiences have meaning beyond the physical world. Many Filipinos believe that God has given them their illness as a destiny and that God’s intention for them includes the potential of death (Doorenbos et al. 2011). The results of the current study revealed a negative relationship between overall spirituality and QoL, along with positive correlations for self-efficacy and life scheme, which may represent the dual function of spirituality, which is to both increase awareness of suffering or mortality and provide existential support.

## Limitations

Notwithstanding the important findings in the study, certain drawbacks must be noted. First, there is the surprising inverse relationship between QoL and spirituality. This could be a result of measuring problems when mixing spirituality subscales, reverse causality, or cultural perspectives on suffering. It is not possible to infer causality because this study was cross-sectional. Also, the study only looked at Filipino women who had breast cancer and was selected using purposive sampling, which has limited how broadly the results might be applied to other similar populations. Future studies may be done taking into account a variety of cultural settings to determine whether the impact of spirituality on quality of life differs among cultures and religions. Also, the relationship between QoL and spirituality should be assessed throughout time to see whether spirituality rises as quality of life falls or whether specific aspects of spirituality have distinct effects on well-being. In addition, studies should compare religious and non-religious forms of spirituality and use longitudinal designs to look at how spirituality and quality of life evolve over the course of the illness. A deeper understanding of the ways in which spirituality interacts with culture, financial load, and coping in breast cancer may also be possible through mixed-methods techniques.

## Conclusion

The study highlights that although spirituality and quality of life had a negative correlation, the subscales of self-efficacy and life scheme had high positive correlations, indicating the complex dimensions of spirituality. Although these results highlight the importance of including spirituality in care, given the limitations, caution must be exercised when making recommendations. Since spirituality may not work the same way for every patient, integrating spiritual treatment should be tailored to each patient’s needs and culturally appropriate. This could entail recognizing spiritual demands as well as family-centered values, Catholic customs, and financial realities for Filipino women. In order to provide comprehensive care that is suited to both cultural and personal settings, healthcare providers can work in conjunction with chaplains, counselors, and community resources.

## Data Availability

The data presented in this study are available on request from the corresponding author. The data are not publicly available due to privacy and ethical restrictions.
